# The Temporal Dynamics of Rumen Microbiota in Early Weaned Lambs

**DOI:** 10.3390/microorganisms10010144

**Published:** 2022-01-11

**Authors:** Shiqin Wang, Jianmin Chai, Guohong Zhao, Naifeng Zhang, Kai Cui, Yanliang Bi, Tao Ma, Yan Tu, Qiyu Diao

**Affiliations:** 1 Key Laboratory of Feed Biotechnology of the Ministry of Agriculture, Institute of Feed Research of Chinese Academy of Agricultural Sciences, Beijing 100081, China; wshq1988@163.com (S.W.); jchai@uark.edu (J.C.); zhaoguoh@foxmail.com (G.Z.); zhangnaifeng@caas.cn (N.Z.); cuikai@caas.cn (K.C.); vetbi2008@163.com (Y.B.); matao@caas.cn (T.M.); tuyan@caas.cn (Y.T.); 2 Anhui Province Key Laboratory of Animal Nutritional Regulation and Health, College of Animal Science, Anhui Science and Technology University, Chuzhou 233100, China; 3 Department of Animal Science, Division of Agriculture, University of Arkansas, Fayetteville, AR 72701, USA

**Keywords:** lamb, early weaning, rumen microbiota, solid diet, rumen development, rumen maturation, weaning stress

## Abstract

Weaning affects the development of ruminal bacteria in lambs during early life. However, the temporal dynamics of rumen microbiota in early weaned lambs is unknown compared to conventionally weaned lambs. In this study, one group was reared with their dams (control, CON) and conventionally weaned at 49 days (d), while the other lambs were weaned at 21 d (early weaning, EW) using starter. Rumen microbial samples collected at 26, 35, and 63 d were used for next-generation sequencing. Here, we found that the abundance and diversity of rumen microbiota in EW were significantly lower at 26 and 35 d than the CON. Linear discriminant analysis Effect Size (LEfSe) analysis was performed to identify the signature microbiota for EW at these three ages. At 26 d, *Prevotella* 7, *Syntrophococcus*, *Sharpea*, *Dialister*, *Pseudoscardovia*, and *Megasphaera* in the rumen of the EW group had greater relative abundances. At 35 d, the *Lachnospiraceae*_NK3A20_group was enriched in CON. On 63 d, *Erysipelotrichaceae*_UCG-002 was abundant in EW. *Syntrophococcus* and *Megaspheaera* in EW lambs were abundant at 26 and 35 d, but kept similar to CON at 63 d. The relative abundance of *Erysipelotrichaceae*_UCG-002 at all-time points was consistently higher in the EW group. In conclusion, early weaning led to a significant decrease in rumen microbiota richness and diversity in the short term. The changes in rumen microbiota are associated with the persistence of weaning stress. The temporal dynamics of relative abundances of *Syntrophococcus*, *Megasphaera*, and *Ruminococcaceae*_UCG-014 reflect the weaning stress over a short period and rumen recovery after early weaning.

## 1. Introduction

Rumen, as a vital organ, plays a critical role in livestock. The structure and function of the rumen develops rapidly in the early stages of life, such as non-rumination, transition, and rumination. Previous studies have shown that early weaning can promote the early development of rumen structure and function [1,2]. For young ruminants, rumen maturation and its microbiota evolution are directly related to health and performance. Many factors influence microbial colonization, such as the dam’s vaginal microbiome and the types of microbes in the surrounding environment [3,4,5]. Feeding modes also affect the direct transmission of bacteria from the mother and the environment to newborns [6]. As rumen develops, its microbial community changes. Moreover, factors including weaning, feeding strategies, solid feed, starter composition, etc. can affect the early establishment and maturation of ruminal microbiota of lambs [2,7,8,9,10]. Weaning is one of the most stressful events in the life of a newborn. Commercial farms often separate ewes and lambs before natural weaning. The early separation results in the breaking of the ewe–lamb bond, with changes occurring in both the physical and social environment at the same time, combining also with the end of suckling and the complete replacement of milk by solid food. The latest research shows that weaning age affects the development of the ruminal bacteria in lambs during early life [2]. Other research has shown that weaning significantly influences the morphological and functional development of the rumen, and bacterial community composition [11].

Since there is currently no standard for early weaning, the effects of early weaning on microbiota are controversial, but it plays a key role in animal performance and development. Until now, there have been two ways of conducting early weaning, including using liquid milk replacement and direct feeding starter. In a previous study, lambs could be weaned at as early as 10 d using a liquid milk replacer [12]. Using a starter to wean lambs is practical in many studies [11,13]. The different weaning strategies result in different levels of animal performance and profit. However, there is no doubt that early weaning will cause a drop in feed consumption and a decrease in the growth rate of lambs in a short period [14,15], and lambs can recover after several weeks [16]. Previous research suggests that early-life nutritional intervention determines the initial rumen microbial community, but the persistence of the effects in later life is weak [17]. Therefore, it has been suggested that alterations of the microbiota for optimizing rumen function may be the most effective approach for rumen development during or immediately following the weaning transition [18]. However, the longitudinal changes in rumen microbiota caused by early weaning are currently unclear.

Based on our previous study, early weaning of lambs at 21 d (early weaning, EW) reduced the average daily gain and affected rumen fermentation parameters in the short term after early weaning compared to conventional weaning (CON) at 49 d when all lambs had the same weaning starter regime [14]. Here, we continue to explore the effects of early weaning using starter on rumen microbiome of lambs weaned at either 21 or 49 days of age. We hypothesized that the composition of rumen microbiota in early weaned lambs is different in the short and long-term. This study could provide a perspective for further understanding of early microbial colonization rules for young ruminants.

## 2. Materials and Methods

### 2.1. Experimental Design, Animal Management, and Sampling

The study was conducted from October to December 2018, at Runlin sheep farm (N 36°82′, E 115°83′) in Liaocheng city, Shandong province, China. The experiment protocol was approved by the Animal Ethics and Humane Animal Care of the Chinese Academy of Agricultural Sciences (protocol#: FRI-CAAS-20180810).

A total of 60 neonatal Hu lambs (48 males and 12 females) with similar birth weights (3.82 ± 0.46 kg) were selected and they were born from double pregnancy. Lambs were randomly assigned into two groups based on body weight at 21 days of age. One group was reared with their dams and conventionally weaned (control, CON) at 49 d, while the lambs in the other group were separated from their dams at 21 d (early weaning, EW). This trial lasted from 21 to 63 d for both CON and EW groups.

From their birthday to 21 d, all lambs (*n* = 60) were reared with ewes (*n* = 12) in 6 well ventilated sheep pens (4 m × 5 m) with controlled temperature and humidity. Each pen had ewes (*n* = 5) and their lambs (*n* = 10, 8 male and 2 female). From 21 d to 63 d, control lambs with their ewe stayed in the same pen, while ewes of EW group were removed and EW lambs were kept in the same pen. All lambs consumed the same starter (21.5% crude protein, and 15.1% NDF) from 7 to 35 d, while another starter (21.5% crude protein, and 18.9% NDF) was provided from 36 to 63 d. All lambs had free access to starter and water during the whole experiment.

Six male lambs per treatment (two lambs from each pen) were randomly selected and sacrificed at 26, 35, and 63 d. The rumen digesta were collected and aliquoted to two tubes. One of tubes was snap-frozen in liquid nitrogen and subsequently stored at −80 °C freezer for further microbial analyses. Another one was stored at −20 °C for analyses of the volatile fatty acids (VFAs) and ammonia nitrogen (NH_3_-N) as described previously [14].

### 2.2. DNA Extraction and 16S rRNA Sequencing

After samples were thawed, the DNA of the rumen content samples was extracted. The extraction procedure followed the instructions of the E.Z.N.A. Stool DNA Kit (Omega Biotek, Norcross, GA, USA). After completed the DNA extraction, 1% agarose gel electrophoresis and spectrophotometry and a Thermo NanoDrop 2000 UV microphotometer (Thermo Scientific, Waltham, MA, USA) were used to detect DNA quality and concentration. Then, we used bacterial DNA as a template, and primers with specific barcode to perform bacterial 16S rDNA gene amplification in the V3–V4 sequencing region. The steps of bacterial 16S rRNA gene sequence amplification are as follows: PCR started with an initial denaturing step at 94 °C for 5 min, followed by 28 cycles at 94 °C for 30 s, 55 °C for 30 s, and 72 °C for 60 s, and a final extension at 72 °C for 7 min. The PCR products were detected using 2% agarose gel electrophoresis. During DNA extraction and PCR step, negative and positive controls were included for quality control. We did not detect any PCR products from these two negative controls on an agarose gel. Therefore, we believe that contamination was minimal and unlikely to have affected biological variation in the different groups. TruSeq^®^DNA PCR-Free DNA Library Construction Kit (Illumina Inc., San Diego, CA, USA) was used for library construction [19]. After the constructed library was quantified by the Qubit Fluorometer (Thermo Fisher Scientific, Waltham, MA, USA) and KAPA Illumina Library Quantification Kits (Roche, Indianapolis, IN, USA), it was sequenced on the Illumina MiSeq-PE300 platform (Illumina Inc., San Diego, CA, USA) and generated 2 × 300 bp paired-end reads.

### 2.3. Bioinformatics and Data Analysis

After original sequencing data was obtained, Trimmomatic (v0.36) (SLIDINGWINDOW:50:20 and MINLEN:120) was used to trim adapters and filter sequences, producing sequences with a phred score over 20 and a length over 120 bp. Then, Pear (v0.9.6) were used to remove the reads with N base exceeding 5%. The chimeras and singletons were detected and removed by Vsearch software (v2.7.1) and high-quality sequences were clustered into operational taxonomic units (OTUs) at a 97% similarity level using the Uparse algorithm in QIIME (Version 1.8.0) [20]. The most frequently occurring sequence in OTUs was selected as the representative sequence of OTUs, and aligned against to GreenGenes bacterial database using the RDP Classifier (Version 2.2) Bayesian algorithm. The microbial sequencing data of this study are available in the NCBI SRA database with the BioProject ID: PRJNA792702.

Samples were normalized to 24,773 sequencing reads, and Qiime software (v.1.8.0) [21] was used for statistical analysis of alpha diversity, including observed-species and Shannon index. The Unifrac distances (both unweighted and weighted) were calculated based on the difference in evolution information between each sample sequence in Qiime software, and R packages were used for visualization of principal co-ordinates analysis (PCoA) [22]. ANOSIM was performed to analyze the differences in community structure between groups.

Linear discriminant analysis Effect Size (LEfSe) analysis was performed to determine the genera differentiated in their relative abundances between the three age groups [23]. Taxa with an LDA Score > 2.5 were considered as exhibiting a significant effect size. To find the relationship between rumen microbiota and fermentation parameters, Spearman correlation analysis was performed. A heat map was used to show the correlations.

## 3. Results

### 3.1. Diversity and Richness of Rumen Microbial Communities

A total of 1,705,188 high-quality sequences were obtained from 36 rumen samples with an average of 47,366 sequences per sample, and 2820 operational taxonomic units (OTUs) were detected based on 97% similarity. With a subsample of 24,733 sequences (the minimum number of reads detected per sample) for each sample, the Good’s coverage (>0.98) revealed that our data provided sufficient sequencing depth to accurately describe the rumen bacterial composition of the lambs used in this study.

**Figure 1:**
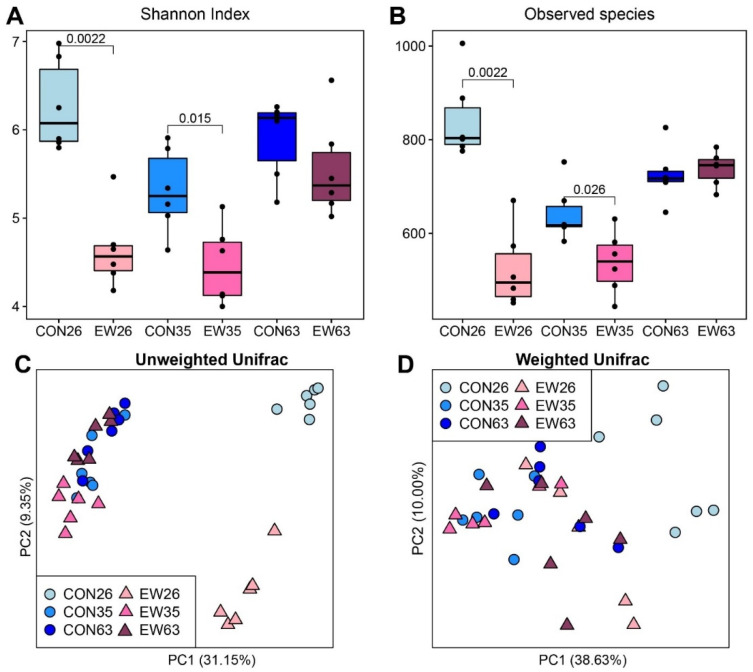
Alpha diversity and similarity of rumen microbiota in control (CON) and early weaned (EW) lambs at 26, 35, and 63 days of age (d). (**A**,**B**): Alpha diversity in the rumen microbial community based on the Shannon index and observed species; (**C**,**D**): The principal coordinate analysis (PCoA) based on the unweighted and weighted Unifrac distances. Each point represents a unique sample. CON26 = control at 26 d; EW = early weaning group at 26 d, the rest can be deduced by analogy.

Compared with the CON group, the abundance and diversity of rumen microbiota in the EW group was significantly lower at 26 and 35 d (*p* < 0.05) as indicated by both Shannon index and the number of observed species, while the differences between the two groups at 63 d were not statistically significant (Figure 1A,B). Then, changes of alpha diversities of rumen microbiota in lambs of different ages were also analyzed. For the CON group, the alpha diversities of the rumen microbiota were significantly reduced (*p* < 0.05) from 26 to 35 d, and increased (*p* < 0.05) from 35 to 63 d (Figure S1). Notably, the number of observed species of the CON groups at 63 d was lower (*p* < 0.05) than at 26 d, although no difference of Shannon index was observed between these two ages. Regarding the EW groups, while no differences of both Shannon Index and the number of observed species were detected between 26 and 35 d, significant increases of alpha diversities at 63 d were observed.

Principal Coordinate Analysis (PCoA) was performed based on unweighted and weighted Unifrac distances (Figure 1C,D). The rumen microbiota of lambs in the EW and CON groups showed distinct clusters on 26 d (ANOSIM, R = 0.88, *p* = 0.003) based on unweighted Unifrac distance, and EW and CON were also distinct at 35 and 63 d (ANOSIM: R = 0.15, *p* = 0.08; R = 0.32, *p* = 0.03). Based on Weighted Unifrac distance, these patterns were also observed, although increased variation within the group. Moreover, age effects on bacterial community structure were observed. For instance, the microbiota of both CON and EW groups at 26 d were distinct compared to other ages.

### 3.2. Bacterial Composition at Different Taxonomical Levels

**Figure 2:**
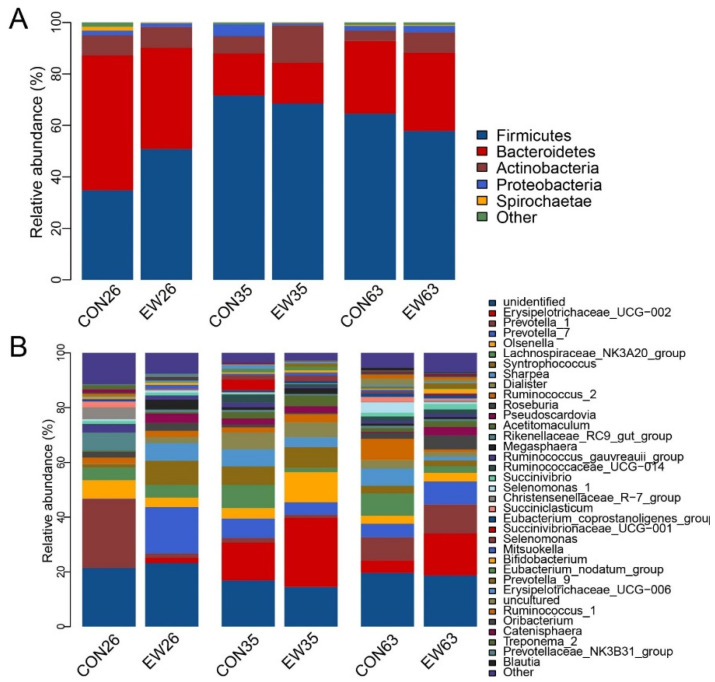
The composition of rumen microbiome at phylum and genus level. (**A**): The composition of rumen microbiome at phylum level; (**B**): The composition of major rumen genera. CON26 = control at 26 d; EW = early weaning group at 26 d. The rest can be deduced by analogy.

A total of 16 phyla of the rumen contents in both CON and EW groups were identified. The main phyla across all samples were *Firmicutes* with the highest relative abundance, followed by *Bacteroidetes*, *Actinobacteria*, and *Proteobacteria*, accounting for over 95% of total reads (Figure 2A). At 26 d, the relative abundance of *Firmicutes* in the CON group was 34.8%, while its relative abundance in the EW group increased to 50.8%. At the same time, the relative abundance of *Bacteroides* in EW was 39.3% compared to its relative abundance in CON with 52.3%. Additionally, *Firmicutes* in both CON and EW groups increased from 26 to 35 d, and subsequently decreased at 63 d. However, the opposite pattern of *Bacteroides* with age was observed.

At the genus level, a total of 208 genera were identified in the rumen contents of all lambs. The top 18 genera had relative abundances more than 1.0%, accounting for 66.74% in total. These bacteria mainly included *Erysipelotrichaceae* UCG-002, *Prevotella* 7, *Prevotella* 1, *Olsenella*, *Lachnospiraceae* NK3A20 group, *Sharpea*, and *Syntrophococcus*, etc. (Figure 2B). Then, we analyzed the ruminal bacteria composition of lambs affected by early weaning at different ages. At 26 d, these genera of *Prevotella* 7, *Syntrophococcus*, *Sharpea*, *Dialister*, *Pseudoscardovia*, and *Megasphaera* in the EW group had greater (*p* < 0.05) relative abundances, while the relative abundances of *Prevotella* 1, *Rikenellaceae*_RC9_gut_group and *Ruminococcaceae*_UCG-014 were higher (*p* < 0.05) in the CON group. On 35 d, the relative abundance of the *Lachnospiraceae*_NK3A20_group in the EW group was reduced significantly (*p* < 0.05) compared with the CON group. At 63 d, the relative abundance of *Ruminococcus*_2 in the rumen of EW group was significantly lower (*p* < 0.05), but *Erysipelotrichaceae*_UCG-002 increased significantly when compared to CON group (Table S1). Additionally, the relative abundance of *Erysipelotrichaceae*_UCG-002 reached its peak at 35 d, while *Prevotella* 1 had the lowest relative abundance at 35 d in the lambs in the EW group.

### 3.3. Bacterial Biomarkers for Early Weaning (EW) at Different Ages

**Figure 3:**
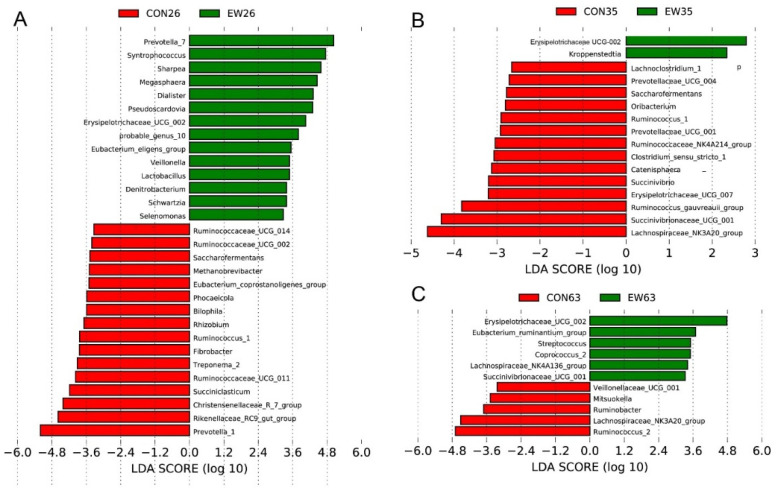
LEfSe analyses of rumen microbiota. LEfSe identified significantly different bacteria at the genus level as differentiating the two groups (control (CON) and early weaned (EW)) at 26 d (**A**), 35 d (**B**) and 63 d (**C**). Genera in this graph were statistically significant (*p* < 0.05) and had an LDA Score > 2.5, which was considered a significant effect size.

To identify the bacterial taxa differentiating CON and EW groups at different ages, Linear discriminant analysis Effect Size (LEfSe) analysis was performed at the genus level. At 26 d, the bacteria with significant differences in the CON group were *Prevotella* 1, *Rikenellaceae* RC9 gut group, and *Christensenellaceae* R 7 group, while the bacterial biomarkers for the EW group were *Prevotella* 7, *Syntrophococcus*, *Sharpea*, *Megaspheaera*, *Pseudoscardovia*, and *Erysipelotrichaceae* UCG-002 (Figure 3A). Upon sampling rumen microbiota at 35 d, *Lachnospiraceae*_NK3A20_group was enriched in the CON group, but *Erysipelotrichaceae* UCG-002 was identified as biomarker for the EW group (Figure 3B). At the end of this trial (63 d), rumen microbiota in lambs of the CON group had high relative abundances of *Ruminococcus* 2 and *Lachnospiraceae*_NK3A20_group, whereas *Erysipelotrichaceae* UCG-002 and *Eubacterium ruminantium group* were over-represented in the EW group (Figure 3C).

### 3.4. Temporal Dynamics of Rumen Microbiota in CON and EW Groups

To better understand how early weaning affects the maturation process of rumen microbiota in early weaned lambs, it is necessary to compare the temporal dynamics of control groups at the same ages. Regarding the longitudinal changes of rumen microbiota in CON groups from 26 to 63 d, LEfSe algorithm was performed to find the biomarkers for each age (Figure S2A). For example, *Prevotella* 1, *Christensenellaceae* R 7 group, and *Erysipelotrichaceae*_UCG-004 were higher at 26 d; *Prevotella* 7, *Erysipelotrichaceae*_UCG-002, *Dialister*, *Pseudoscardovia*, and *Ruminococcaceae*_UCG-014 were enriched at 35 d; and *Sharpea*, *Megaspheaera*, and *Erysipelotrichaceae* UCG-009 were abundant at 63 d. For rumen microbiome of early weaning lambs, bacterial biomarkers for each age were also identified. At 26 d, *Syntrophococcus*, *Megaspheaera*, *Selenomonas*_1 and *Erysipelotrichaceae*_UCG-004 as the EW signatures were also classified. At 35 d, *Erysipelotrichaceae* UCG-002, *Acetitomaculum*, and *Selenomonas* were enriched in the ruminal community. At 63 d, the abundant genera included *Prevotella* 1, *Ruminococcaceae*_UCG-014, *Roseburia*, *Succinivibrio*, *Prevotella* 9, *Syntrophococcus*, and *Christensenellaceae* R 7 group.

**Figure 4:**
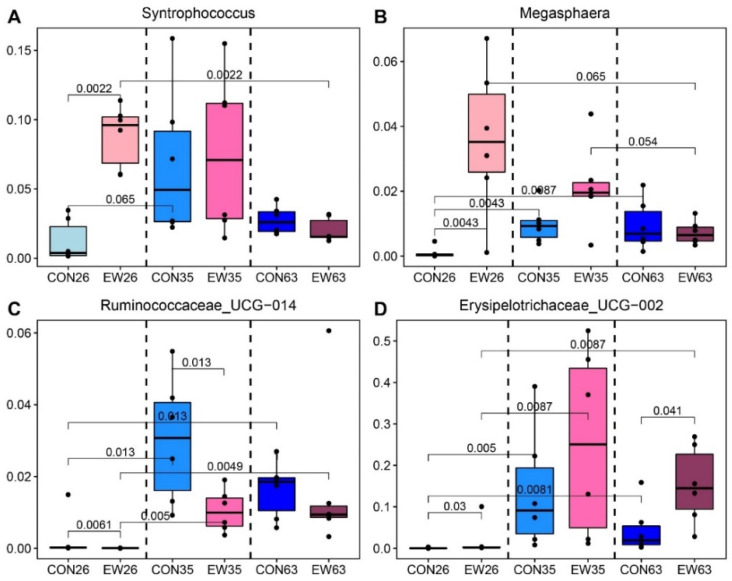
Temporal dynamics of the bacterial biomarkers for lambs changed with ages. The important bacterial biomarkers, including *Syntrophococcus* (**A**), *Megasphaera* (**B**), *Ruminococcaceae*_UCG-014 (**C**) and *Erysipelotrichaceae* UCG-002 (**D**), showed different patterns of temporal dynamics in control (CON) and early weaned (EW) groups. CON26 = control at 26 d; EW = early weaning group at 26 d. The rest can be deduced by analogy.

To further understand that the bacterial biomarkers for both CON and EW groups changed with ages, the corresponding genera were selected to create a better visualization. For *Syntrophococcus* and *Megasphaera* identified as EW biomarkers, their relative abundances in the CON and EW groups had different patterns changed with ages (Figure 4A,B). In other words, the differences of their relative abundances between CON and EW deceased with age, which is consistent with the results of diversities. *Ruminococcaceae*_UCG-014 were higher in the CON compared to the EW group, and its relative abundance was higher during 35 and 63 d (Figure 4C). *Erysipelotrichaceae* UCG-002 was consistently higher in EW group from 26 to 63 d and its relative abundances in both CON and EW groups increased with ages (Figure 4D).

### 3.5. Rumen Bacteria Associated with Fermentation Parameters

**Figure 5:**
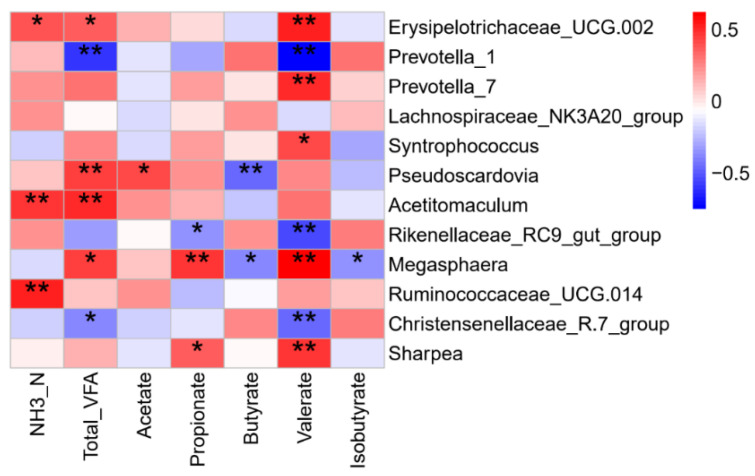
Spearman correlation analysis of ruminal microbiota and rumen fermentation parameters. Color represents the correlation coefficient, with red representing a positive correlation and blue denoting a negative correlation. * *p* < 0.05 and ** *p* < 0.01.

The data of rumen fermentation parameters were obtained from our previous study [14]. Briefly, the concentration of Total VFA, molar proportion of acetate and propionate in the EW group increased at 5 days after early weaning. A number of significant correlations were found between the rumen microbiota and fermentation parameters (Figure 5). For example, rumen ammonia concentration was positively correlated with the relative abundances of *Erysipelotrichaceae*_UCG-002, *Acetitomaculum*, and *Ruminococcaceae_UCG-014*; acetate had a positive correlation with *Pseudoscardovia*; propionate was negatively correlated with the *Rikenellaceae*_RC9_gut_group but positively correlated with *Megasphaera* and *Sharpea*; butyrate was negatively correlated with *Pseudoscardovia* and *Megasphaera*.

## 4. Discussion

### 4.1. The Diversity and Richness of Rumen Microbiota Influenced by Early Weaning

The diversity and richness index are usually used to evaluate the stability of an ecosystem. Our results show that early weaning caused the decreased richness and diversity of rumen microbiota of lambs, and its impacts mainly happened on 5 and 14 days after weaning at 21 d. At the same time, we observed a significant difference in beta diversity of the rumen microbiota between the EW and CON lambs on 26 d. The dissimilarity of the structure of the rumen microbiota between the two groups decreased on 42 days after early weaning. These results suggest that early weaning changed the process of colonization of the rumen microbiota of lambs in a short period, while the diversity and structure of rumen microbial communities could recover at 42 days after weaning. These results partially differ from previous research which found that the alpha diversity and beta diversity between weaning and post-weaning for lambs receiving the strategy of artificial feeding with milk replacer and weaning at 30 d were not significantly different when compared to controls [2]. Another study of the same feeding strategy showed that alpha diversity was not significantly influenced between pre- and post-weaning whether or not lambs were weaned at 21 or 35 d [13]. Differences with reported results may be related to different feeding strategies. One of the studies pointed out that supplementary feeding with alfalfa before weaning could avoid changes in the rumen microbial diversity and abundance before and after weaning [8]. Feeding mode is an important driver of early microbial colonization, influencing the structure and function of lambs’ gut microbiome [6]. Our study is based on the feeding mode of breastfeeding, which may cause higher stress in a short period. However, at 42 days after early weaning, the rumen microbiome in early weaned lambs could reach the same level as control lambs. This suggests that the mode of weaning lambs at 21 days of age and feeding with solid feed may be feasible for actual production.

After the birth of young ruminants, the rumen microbiota is in a process of constant change with age and the maturation of rumen [24,25]. So, we further studied the diversity and richness changes with age of the rumen microbiota of lambs in the two groups. In the CON group, the diversity and richness of bacterial communities increased with age, which is in accordance with the results of previous studies [7,11,25]. This is mainly related to the continuous changes in lambs’ breast milk and starter intake to continuously adjust and adapt to rumen fermentation substrates. At 63 d, the diversity and abundance of rumen microorganisms increased, indicating that the rumen microbiota was established more completely to better adapt to the digestion of solid feed. In EW group, the rumen microbial diversity and richness of lambs at 63 d were higher than at 26 and 35 d, which is consistent with the previous research reported [2,11]. As we know, for early weaned lambs, the diet was directly converted from breast milk to solid feed and lambs’ feed intake of starer after weaning increased sharply. Previous studies showed that the feeding of starter is an important factor affecting the colonization of young ruminants before ruminating [8,9,26]. The above results indicate that early nutritional intervention could affect the initial microbial formation in the rumen, and the influence of weaning on the rumen community is the most obvious [27]. Early weaning stress has adverse effects on lambs’ growth and health [14], and impacts the richness and diversity of rumen microbiota. So, it is necessary to know the short-term and long-term effects of early weaning stress on the rumen microbiota of lambs, and how the rumen microbiotas change during the stress transition and recovery periods.

### 4.2. The Compositions of Rumen Microbiota Are Associated with Early Weaning

At the phylum level, the rumen microbiota of lambs in control and early weaning groups are mainly *Firmicutes*, *Bacteroidetes*, *Actinobacteria*, and *Proteobacteria*, which is consistent with previous research results in lambs [8,13,24]. This study shows that early weaning affected the composition of microbes in the rumen of lambs in the short-term. As in the phylum, after early weaning, the relative abundance of *Firmicutes* in the rumen significantly increased, while the percent of *Bacteroides* significantly decreased on day 26 of age. After early weaning, solid feed was increased significantly. The increases in relative abundance of *Firmicutes* are related to high grain feeding, energy harvesting or feed efficiency [28,29,30]. Studies on dairy cows have shown that high grain feeding increased the relative abundance of *Firmicutes* [30]. However, one study showed no significant difference in relative abundance of any of the top four bacterial phyla between different ages [2] and this may be related to different feeding strategies or weaning age. Our previous research found that there were significant differences between the two different feeding strategies of ewe-lamb breastfeeding or artificial feeding [6]. This study was based on the production mode of lambs in most farms where the lambs are breastfed until final weaning.

To further understand the effects of early weaning on rumen microbiota, the signature genera for two groups were identified. Our results show that early weaning mainly influenced the microbial composition at 5 days after weaning. At 26 d (5 days after early weaning), the composition of the microbiota changed significantly. The relative abundance of the genera, such as *Prevotella* 7, *Sharpea*, *Syntrophococcus*, *Dialister*, and *Megasphaera*, increased significantly in early weaned lambs. As we know, *Prevotella* is one of the dominant genera within phylum *Bacteroidetes* in the rumen, which is consistent with the results on lambs [31]. It can degrade and utilize starch and plant cell wall polysaccharides, such as xylan, and pectin, in the rumen for fermentation, and the products are acetic acid, succinic acid, and propionic acid [32]. *Prevotella* also could utilize lactic acid and convert it into propionic acid [33]. Thus, an increase in the relative abundance of *Prevotella* is beneficial for ruminants that consume a high concentrate diet [34,35]. *Syntrophococcus*, *Dialister*, *Megasphaera*, and *Sharpea* are all members of *Firmicutes* that could digest various carbohydrates to produce short chain fatty acids [36]. The increased relative abundances of these bacteria result in the improved ability to digest carbohydrates with increasing solid feed intake in the early weaned lambs after early weaning. An increase of *Dialister* has been associated with hyposalivation [37], which may play a role in altering the buffering capacity of the rumen and fluid turnover. *Sharpea* is the lactate-producer [38], and *Megasphaera* can converse the lactate to butyrate [39]. Thus, *Sharpea* was accompanied by a corresponding increase in the percentage of *Megasphaera*, which increased butyrate production. Overall, the increased relative abundance of these genera in the rumen after weaning can promote digestion and utilization of carbohydrates. After early weaning, we found a significant decrease in the relative abundance of *Rikenellaceae*_RC9_gut_group and *Ruminococcaceae*_UCG-014. The family *Rikenellaceae* is found to be involved in the degradation of structural carbohydrates [40,41]. One study reported that cows fed high-starch diets with supplemental oil decreased the relative abundance of *Rikenellaceae*_RC9 [41].

During the recovery transition period (14 days after early weaning), only a small number of bacteria were affected after early weaning. *Erysipelotrichaceae* UCG-002 and *Selenomonas*_1 were enriched in the ruminal community of EW lambs. Compared with the previous stage, the biggest change was in *Erysipelotrichaceae* UCG-002, which became the dominant bacteria, which was associated with the rumen total volatile fatty acids concentration. *Selenomonas*_1 is a non-spore forming anaerobic bacteria that plays an essential role in the production of volatile fatty acids, especially propionic acid in the rumen [42]. At this stage, weaning stress decreased after the adaptation of starter intake, as we observed that the starter intake increased significantly, and the rumen developed rapidly in our previous study [14].

On day 42 of age after early weaning, the differences between CON and EW groups became smaller. *Erysipelotrichaceae*_UCG-002 was still the dominant bacteria in the early weaning group, while *Rumenococcus* 2 was the dominant bacteria in the control group. At this stage, all lambs were weaned and fed the same solid feed, resulting in a similar concentration of rumen fermented substrates. When challenged by a microbial community different to the rumen conditions of the host animal, the rumen can successfully rebuild its own microbiota [43]. This indicated that a time-dependent effect on some bacteria before stabilization may occur with age. The persistent changes of the same bacterial species in the rumen are the result of adapting to the composition of carbohydrates in the transition from liquid feed to a starter.

Similar research results also show that lambs weaned at 21 or 35 d did not affect the rumen microbiota and rumen fermentation parameters at 50 d [13]. The difference in rumen microbiota of young animals is not affected by weaning time or weaning method, but is mainly related to whether weaning or not. One study on calves has shown that weaning had a significant impact on the composition and structure of the rumen microbiota, while weaning methods have less impacts on it [44]. Maternal separation at weaning immediately shifted the composition of the gut microbiota in all animals, revealing fitness differences among species. It is likely that these differences are linked to maternal separation-associated stress. Similarly, O’Mahoney et al. reported that feces of adult mice that had undergone maternal separation for 3 h per day from postnatal days 2–12 presented an altered microbiota composition when compared with the nonseparated control animals [45]. However, the impacts of milk withdrawal on rumen microbiota composition at weaning are less probable and still need further study.

### 4.3. The Temporal Dynamics of Rumen Microbiota after Early Weaning

Compared with weaning, there are higher numbers of bacterial genera which changed with ages. Thus, the temporal dynamics of the bacterial biomarkers for early weaned lambs were investigated, which could help us understand the persistent effects of early weaning. Relative abundance of *Syntrophococcus* was lower in EW lambs at 26 d, but retained similar relative abundance with controls at other ages. *Syntrophococcus* is involved in the utilization of non-fibrous carbohydrates and produces acetic acid [46]. After early weaning, the percent of *Syntrophococcus* increased, which is similar to the results of the recent study on calves [47]. The relative abundance of *Syntrophococcus* decreased at 63 d in both groups compared to 35 d, which may be due to ages and diet. *Megasphaera* is a lactic acid-utilizing bacterium. The proportion of *Megasphaera* in the early weaning group is higher than that of the control group at 26 and 35 d, which proves its relationship with early weaning and starter intake. Research showed that *Megasphaera* is an ecologically important rumen bacterium that metabolizes lactate and relieves rumen acidosis induced by a high-grain-diet [48]. Our results showed that *Megasphaera* was positively correlated with the concentration of propionate, valerate, and total VFA while negatively correlated with butyrate, which reflects the importance of *Megasphaera*. Previous studies on lambs showed that starter feeding promoted the increase of the relative abundance of *Megasphaera* and would be beneficial for weaning of starter-fed lambs [49,50]. After early weaning, lambs only consume solid feed with high grains provide conditions for *Megasphaera* growth, while its relative abundance became similar to controls due to disappearance of the weaning stress. *Ruminococcaceae*_UCG-014 changed with age in the CON compared to EW group. *Ruminococcaceae* is an important fibrolytic bacteria in the guts of mammals [51]. One study has shown that increasing the level of fiber in the diet will lead to *Ruminococcaceae*_UCG-014 growth [52]. In the current study, the relative abundance of *Ruminococcaceae*_UCG-014 increased with increasing age. Moreover, *Ruminococcaceae*_UCG-014 were positively correlated with rumen ammonia concentration. This shows that the ability of lambs to digest fiber and protein enhanced with age. The percent of *Erysipelotrichaceae*_UCG_002 also increased after early weaning, especially in early weaned rumen, and was positively correlated with the concentration of total VFA and valerate. *Erysipelotrichaceae*_UCG_002 belongs to the family of *Erysipelotrichaceae* of *Firmicutes* member which was previously reported to be associated with VFA synthesis [53]. Therefore, *Erysipelotrichaceae* may promote cholesterol production and accumulation [54], which suggests it may be related to increased starter intake after early weaning. Unfortunately, the function of *Erysipelotrichaceae*_UCG_002 is still elusive and thus requires an in-depth exploration in the future.

## 5. Conclusions

Early weaning could cause a significant decrease in richness and diversity in rumen microbiota in the short-term, and lead to corresponding shifts in the composition of rumen microbiota. At 42 days after early weaning, its effects on rumen microbial composition decline. The temporal dynamics of relative abundances of *Syntrophococcus*, *Megasphaera* and *Ruminococcaceae*_UCG-014 reflect the weaning stress over a short period and rumen recovery after early weaning. However, the persistent effects of early weaning may have impacts on the maturated rumen considering the higher relative abundances of *Erysipelotrichaceae*_UCG_002 in early weaned lambs at the end of this trial. Overall, this study demonstrates the temporal dynamics of rumen microbiota in early weaned lambs, which allow us to better understand the effects of early weaning.

## Data Availability

All data are contained within the article or Supplementary Materials.

## Supplementary Materials

The following are available online at https://www.mdpi.com/article/10.3390/microorganisms10010144/s1, Table S1: Differences in the composition of rumen microbial taxon of lambs among different; Figure S1: Alpha diversities of rumen microbiota in lambs; Figure S2: The bacterial biomarker identified by LEfSe for each age in CON and EW groups.
