# Supplementary material for: The Temporal Dynamics of Rumen Microbiota in Early Weaned Lambs
Source: Microorganisms. 2022 Jan 11;10(1):144. doi: 10.3390/microorganisms10010144 (PMC8779368; doi:10.3390/microorganisms10010144)
Supplement: Supplementary file 1 [file microorganisms-10-00144-s001.zip › microorganisms-1509622-supplementary.pdf]

The Temporal Dynamics of Rumen Microbiota in Early Weaned Lambs

Shiqin Wang [1,2,†], Jianmin Chai [1,3,†], Guohong Zhao [1,2], Naifeng Zhang [1], Kai Cui[1], Yanliang Bi [1], Tao Ma [1,†]Yan Tu [1], Qiyu Diao [1,*]

[1] Feed Research Institute, Chinese Academy of Agricultural Sciences/Key Laboratory of Feed Biotechnology of the Ministry of Agriculture and Rural Affairs, Beijing 100081, China.
wshq1988@163.com (S.W.); jchai@uark.edu (J.C.); zhaoguoh@foxmail.com (G.Z.); zhangnaifeng@caas.cn (N.Z.); cuikai@caas.cn (K.C.); vetbi2008@163.com (Y.B.); matao@caas.cn (T.M.); tuyan@caas.cn (Y.T.); diaoqiyu@caas.cn (Q.D.).

[2] College of Animal Science, Anhui Science and Technology University /Anhui Province Key Laboratory of Animal Nutritional Regulation and Health, Fengyang 233100, China.

[3] Department of Animal Science, Division of Agriculture, University of Arkansas, Fayetteville, AR, 72701, USA.

† Shiqin Wang and Jianmin Chai contribute equally to the article.

* Correspondence: diaoqiyu@caas.cn; Tel.: +86-010-8210-6055

Table S1: Differences in the composition of rumen microbial taxon of lambs among different
Figure S1： Alpha diversities of rumen microbiota in lambs
Figure S2: The bacterial biomarker identified by LEfSe for each age in CON and EW groups

Table S1. Differences in the composition of rumen microbial taxon of lambs among different groups (relative abundance above 1.0%)

	d 26		SEM	*P*-values	d 35		SEM	*P*-values	d 63		SEM	*P*-values
	CON	EW			CON	EW			CON	EW		
g__Erysipelotrichaceae_UCG-002	0.06	1.86	0.83	0.024	13.77	25.27	5.51	0.337	4.52	15.32	2.71	0.037
g__Prevotella_1	25.08	1.62	4.59	0.004	1.74	1.11	0.62	0.149	8.49	10.65	2.93	1.000
g__Prevotella_7	0.16	16.91	4.16	0.004	7.05	4.52	1.33	0.873	4.94	8.36	1.44	0.423
g__Olsenella	6.76	3.52	1.84	1.000	3.81	11.06	2.44	0.337	2.94	3.20	0.91	0.109
g__Lachnospiraceae_NK3A20_group	4.62	4.61	1.64	0.200	8.52	1.60	1.41	0.007	8.18	2.47	1.43	0.078
g__Syntrophococcus	1.24	8.83	1.26	0.004	6.73	7.52	1.55	0.631	2.75	2.02	0.28	0.078
g__Sharpea	0.10	6.39	1.97	0.003	6.19	3.58	1.43	0.150	6.31	1.39	1.45	0.055
g__Dialister	0.00	2.35	0.58	0.003	6.26	5.50	0.87	0.262	3.10	1.72	0.53	0.200
g__Ruminococcus_2	2.33	2.18	0.64	0.631	1.80	2.98	0.96	0.470	7.76	0.89	1.80	0.025
g__Roseburia	2.10	2.85	1.24	0.423	1.02	0.50	0.14	0.065	2.15	5.16	1.48	0.423
g__Pseudoscardovia	0.01	3.50	1.33	0.004	2.19	2.35	1.19	0.109	0.34	3.05	1.44	0.377
g__Acetitomaculum	0.37	0.56	0.12	0.522	2.43	3.96	1.36	0.522	1.52	2.19	0.41	1.000
g__Rikenellaceae_RC9_gut_group	6.59	0.83	1.53	0.010	0.74	0.50	0.20	0.150	0.71	0.72	0.14	0.631
g__Megasphaera	0.10	3.60	0.69	0.007	0.98	2.14	0.33	0.109	0.95	0.72	0.17	0.749
g__Ruminococcus_gauvreauii_group	2.78	1.55	0.76	0.873	1.60	0.51	0.35	0.055	0.86	1.06	0.23	0.337
g__Ruminococcaceae_UCG-014	0.27	0.01	0.12	0.007	3.01	1.05	0.47	0.037	1.63	1.73	0.45	0.522
g__Succinivibrio	1.13	1.06	0.48	0.522	0.43	0.14	0.07	0.065	1.38	1.95	0.56	0.631
g__Selenomonas_1	0.64	0.79	0.23	0.423	0.13	0.04	0.04	0.197	3.80	0.66	1.54	0.128

The *P* values were calculated by using Kruskal–Wallis test. CON=lambs weaned at day 49 as conventional weaned group; EW=lambs weaned at day 21 as early weaned group. In the same row, P＜0.05superscripts mean significant difference, SEM = standard error of the mean.

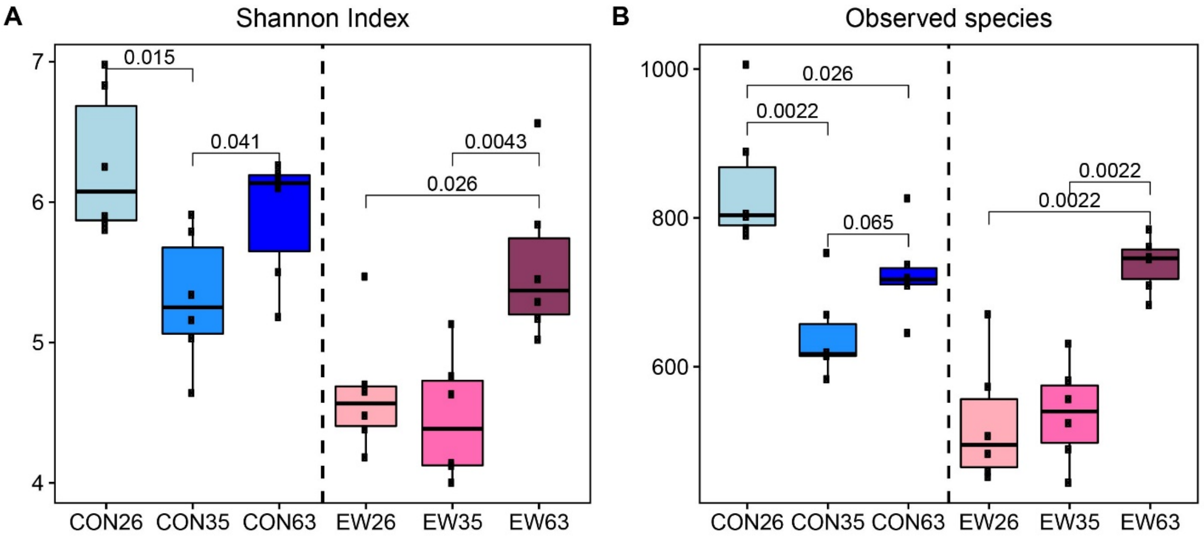

Figure S1. Alpha diversities of rumen microbiota in lambs. CON=lambs weaned at day 49 as conventional weaned group; EW=lambs weaned at day 21 as early weaned group. Microbial diversities and structure of CON and EW groups at 26, 35 and 63 days of age (d). Alpha diversity in the rumen microbial community based on the Shannon Index(A): and Observed species (B). CON26= control at 26 d; EW= early weaning group at 26 d, the rest can be deduced by analogy.

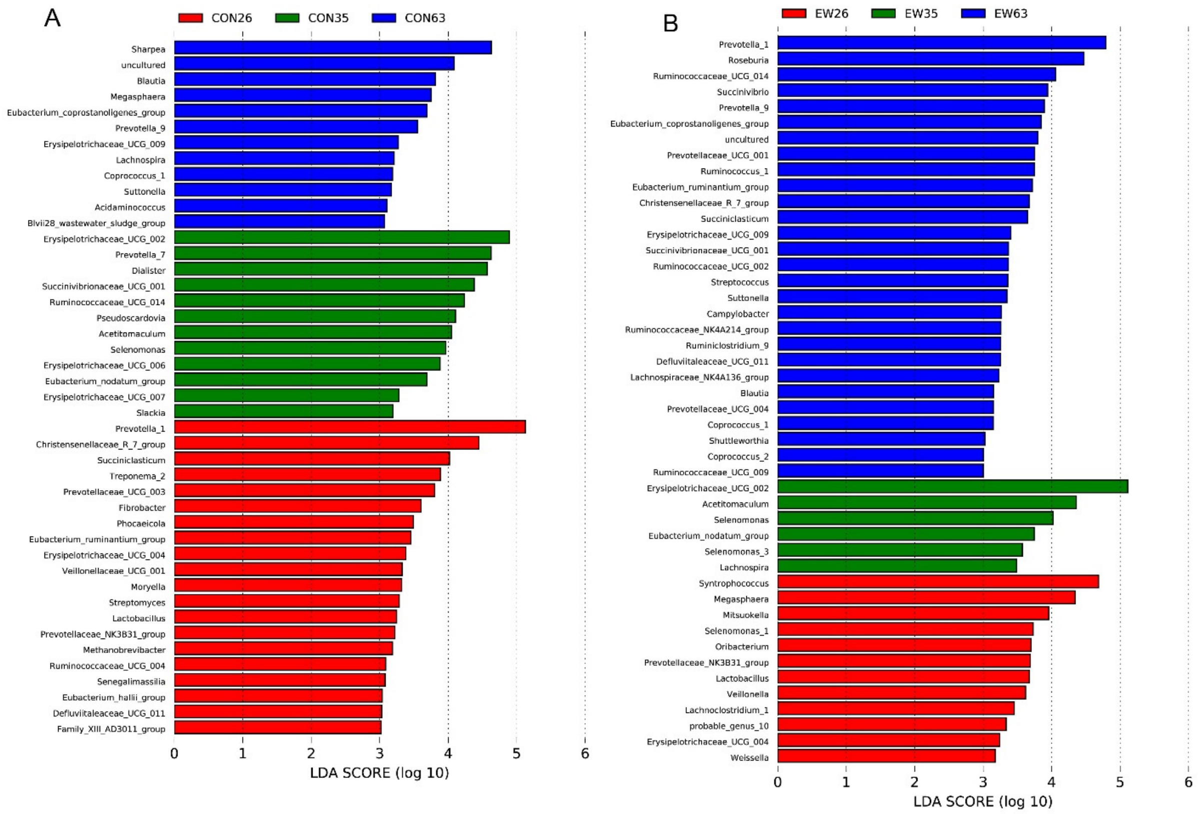

Figure S2. The bacterial biomarker identified by LEfSe for each age in CON and EW groups. LEfSe analyses of rumen microbiota. CON=lambs weaned at day 49 as conventional weaned group; EW=lambs weaned at day 21 as early weaned group. The bacterial biomarker identified by LEfSe for each age in CON (A) and EW groups (B). Genera in this graph were statistically significant ($p < 0.05$) and had an LDA Score >2.5, which was considered a significant effect size.
